# The haemostatic effect of deep-frozen platelets versus room temperature-stored platelets in the treatment of surgical bleeding: MAFOD—study protocol for a randomized controlled non-inferiority trial

**DOI:** 10.1186/s13063-022-06739-2

**Published:** 2022-09-24

**Authors:** Tim W. H. Rijnhout, Femke Noorman, Robert A. van der Horst, Edward C. T. H. Tan, Victor V. A. Viersen, Oscar J. F. van Waes, Leo M. G. van de Watering, B. L. S. Borger van der Burg, Jaap J. Zwaginga, Michael H. J. Verhofstad, Rigo Hoencamp

**Affiliations:** 1 Department of Surgery, Alrijne Hospital, Simon Smitweg 1, 2353 GA Leiderdorp, The Netherlands; 2 Trauma Research Unit Department of Surgery, Erasmus MC, University Medical Center Rotterdam, 3000 CA Rotterdam, The Netherlands; 3 Military Blood Bank, Ministry of Defence, 3584 EZ Utrecht, The Netherlands; 4 Department of Surgery, Radboud University Medical Centre, Nijmegen, The Netherlands; 5 Department of Anesthesiology, Amsterdam University Medical Centre, Location AMC, Amsterdam, The Netherlands; 6 Centre for Clinical Transfusion Research, Sanquin Research, Leiden, The Netherlands; 7 Jon J van Rood Center for Clinical Transfusion Research, Sanquin-Leiden University Medical Center, Leiden, The Netherlands; 8 Department of Haematology, Leiden University Medical Centre, Leiden, The Netherlands; 9 Defence Healthcare Organization, Ministry of Defence, 3584 AB Utrecht, The Netherlands; 10 Department of Surgery, Leiden University Medical Centre, 2333 ZA Leiden, The Netherlands

**Keywords:** Transfusion, Cryopreservation, Trauma, Bleeding, Haemorrhage, Platelets, Resuscitation, Injury, Blood, Coagulopathy

## Abstract

**Background:**

The Netherlands Armed Forces have been successfully using deep-frozen (− 80 °C) thrombocyte concentrate (DTC) for the treatment of (massive) bleeding trauma patients in austere environments since 2001. However, high-quality evidence for the effectiveness and safety of DTCs is currently lacking. Therefore, the MAssive transfusion of Frozen bloOD (MAFOD) trial is designed to compare the haemostatic effect of DTCs versus room temperature-stored platelets (RSP) in the treatment of surgical bleeding.

**Methods:**

The MAFOD trial is a single-blinded, randomized controlled non-inferiority trial and will be conducted in three level 1 trauma centres in The Netherlands. Patients 12 years or older, alive at hospital presentation, requiring a massive transfusion including platelets and with signed (deferred) consent will be included. The primary outcome is the percentage of patients that have achieved haemostasis within 6 h and show signs of life. Haemostasis is defined as the time in minutes from arrival to the time of the last blood component transfusion (plasma/platelets or red blood cells), followed by a 2-h transfusion-free period. This is the first randomized controlled study investigating DTCs in trauma and vascular surgical bleeding.

**Discussion:**

The hypothesis is that the percentage of patients that will achieve haemostasis in the DTC group is at least equal to the RSP group (85%). With a power of 80%, a significance level of 5% and a non-inferiority limit of 15%, a total of 71 patients in each arm are required, thus resulting in a total of 158 patients, including a 10% refusal rate. The data collected during the study could help improve the use of platelets during resuscitation management. If proven non-inferior in civilian settings, frozen platelets may be used in the future to optimize logistics and improve platelet availability in rural or remote areas for the treatment of (massive) bleeding trauma patients in civilian settings.

**Trial registration:**

ClinicalTrials.gov NCT05502809. Registered on 16 August 2022.

**Supplementary Information:**

The online version contains supplementary material available at 10.1186/s13063-022-06739-2.

## Background and rationale

Major haemorrhage (defined as total loss of circulating blood volume within 24 h, 50% in less than 3 h or bleeding in excess of 150 mL/min) is still a leading cause of preventable death in both military and civilian settings. In case of survival, it often results in high morbidity and a decrease in quality of life [1]. Simultaneously with haemorrhage control, circulating volume should be repleted with blood or blood components (red blood cells (RBC), plasma, room temperature (22 °C)-stored platelets (RSP)) [2–4]. However, when stored in liquid form, the shelf life of blood components is limited to up to 35 days for units RBC and whole blood, 7 days for RSPs and 5 days for fresh frozen plasma (FFP) (stored at 4 °C after thaw). Due to the short shelf life of RSP, it cannot be used for hospitals in austere environments that require multiple days of transport to restock. Possible alternatives to RSP are a collection of apheresis platelets or fresh whole blood (FWB) from donors on location (walking blood banks) or buddy transfusion. To avoid dependence on this walking blood bank, the Netherlands Armed Forces (NLAF) implemented deep-frozen blood RBCs, plasma and platelets (DTC) in 2001. These deep-frozen products are derived from fully tested donors in the home country and are subsequently frozen and stored at − 80 °C by the Dutch Military Blood Bank (MBB) [5, 6]. This − 80 °C storage temperature prolongs the shelf life from days to multiple years and allows long-distance transports with the use of dry ice in insulated shipping containers and subsequent long on-site storage prior to use. This concept has been shown to optimize stock management and reduce product waste due to expiration [6].

DTCs can be stored for at least 4 years at − 80 °C [7]. Apart from this logistical advantage, in vitro product characteristics of DTCs differ from RSPs. Indeed, DTCs show impaired aggregation in classic aggregation tests and clot strength but accelerated clot formation and thrombin formation as compared to other platelet products [8–10]. These differences are likely due to platelet activation and release of platelet-derived microparticles after the thaw [11]. The use of DTCs in several clinical studies has not led to effectivity or safety issues in trauma [12], thrombocytopenic [11, 13] or cardiopulmonary bypass [9] patients and in rats [12]. In the currently introduced multi-centre, single-blinded, randomized controlled non-inferiority trial, the objective is to compare the haemostatic effects of DTCs with the currently predominantly used RSPs in the treatment of surgical bleeding and to show that DTCs have at least similar haemostatic effects as compared to RSP.

## Design and methods

### Study setting

**Fig. 1:**
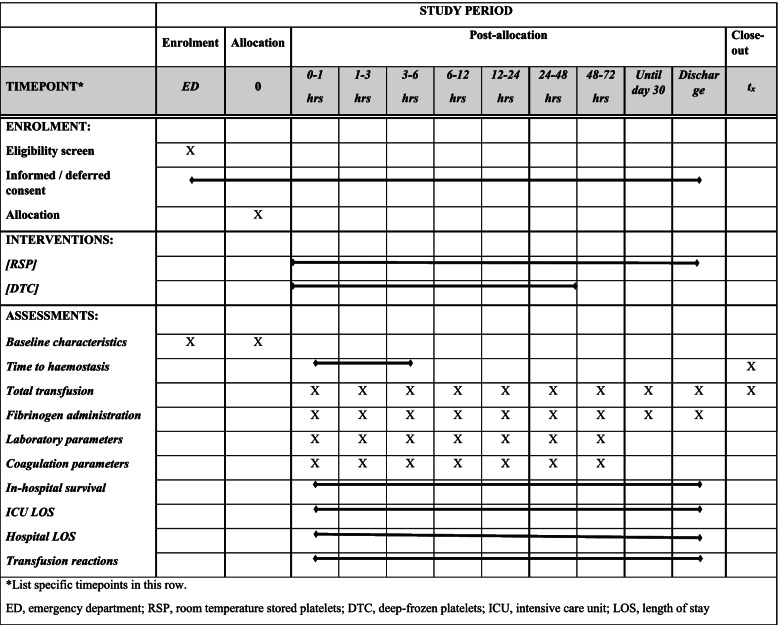
SPIRIT schedule of enrolment, interventions and assessments

The *MAssive transfusion of Frozen bloOD* (MAFOD) trial is a multi-centre, single-blinded, randomized controlled non-inferiority trial and will be conducted in three level 1 trauma centres in The Netherlands. The protocol is written in accordance with the Standard Protocol Items: Recommendations for Interventional Trials (SPIRIT) Additional file 1, and the schedule of enrolment, interventions and assessments is shown in Fig. 1.

### Eligibility criteria

This study focuses on patients suspected and treated for major haemorrhage (traumatic and vascular surgical bleeding). Patients 12 years or older, alive at hospital presentation requiring massive transfusion including platelets and signed (deferred) consent will be included. When patients are deceased prior to obtaining consent, data collected up to that point will be used. Patients receiving prehospital transfusion with blood products or additional medication (e.g. fibrinogen concentrate) will also be included. Informed consent will be requested at the earliest available moment.

### Intervention and control

The patient will receive usual care while participating in this study. Patients randomized to the intervention group will receive thawed DTC (re-suspended in cold stored deep-frozen plasma (DFP)) during the first 48 h after randomization. It is possible for clinicians to submit a request at any time to immediately transfuse RSPs. The control group will receive RSP in platelet additive solution. The patient timeline is summarized in Fig. 1.

### Outcomes

The primary outcome is the percentage of patients that achieved haemostasis at 6 h and show signs of life. Time to haemostasis is defined as the time in minutes from arrival to the last transfused blood component followed by a 2-h transfusion-free period of units RBC. All study parameters are listed in Table 1 and described in ClinicalTrials.gov.

**Table 1:** Outcomes

Outcome measure	Interval	Unit
Haemostasis (primary)	6 h	%
Time to haemostasis		min
Transfused blood components	0–1, 1–3, 3–6, 6–12, 12–24, 24–48, 48–72 h; 72-h 30 days and total	Units
Fibrinogen administration	0–1, 1–3, 3–6, 6–12, 12–24, 24–48, 48–72 h; 72-h 30 days and total	g
Laboratory parameters	0–1, 1–3, 3–6, 6–12, 12–24, 24–48, 48–72 h	• Haemoglobin (mmol/L) • Haematocrit (L/L) • Platelet count (× 10^9^/L)
Coagulation parameters	0–1, 1–3, 3–6, 6–12, 12–24, 24–48, 48–72 h	• Fibrinogen (Clauss) (g/L) • INR • aPTT (s)
In-hospital survival (different time intervals)		Yes/no
Hospital LOS		Days
ICU LOS		Days
Occurrence of transfusion reactions		Yes/no

*INR* international normalized ratio, *aPTT* activated partial prothrombin time, *LOS* length of stay, *ICU* intensive care unit, *n.a.* not applicable

### Sample size calculation

We assume that the percentage of patients in the DTC group that will achieve haemostasis will be at least equal to the RSP group (85%). This percentage is based on the results of the viscoelastic haemostatic assay augmented protocols for major trauma haemorrhage (ITACTIC) trial which included trauma patients with clinical signs of bleeding and massive transfusion protocol (MTP) activation [13]. Sample size calculation was performed using www.sealedenvelope.com with a binary outcome. It is estimated that with a power of 80% and a significance level of 5% and a non-inferiority limit of 15%, a total of 71 patients in each arm are required. Taking a 10% dropout into account, this will result in a total of 158 patients. Presentations will be held frequently to create awareness amongst clinicians, so that the sample size will be reached.

### Allocation

The randomization scheme will be stratified into two groups (DTC and RSP). A blocked randomization with randomly chosen block sizes that remain hidden from the researchers will be determined by a scientist not associated with the study. This person will also determine the randomization sequence which will be kept hidden from all personnel that is in any way involved in the selection or inclusion of the subjects. The codes of the randomization sequence will be noted on forms along with an instruction to prepare either room RSP or DTC. These forms will be put into opaque envelopes prior to the start of the study.

The actual randomization will be performed in the blood transfusion laboratory. Because of the hyper-acute setting in which this study is conducted, the use of online randomization would lead to an unacceptable delay. The randomization process will be initiated by a laboratory technician after an MTP package including platelets is requested. The unique number the envelope contains will be the identifier for all study forms related to the DTC preparation and haemovigilance data registration of the MBB. If the envelope contains the text ‘room temperature-stored platelets’, the normal process of blood product issuing will be initiated.

After randomization to the intervention group, the patient will receive DTCs for 48 h when platelets are needed. After 48 h, the patient will receive RSPs when required. An automatic pop-up in the electronic will prevent patients from receiving the product of the opposite arm.

In case of problems with randomization, the standard preparation of thrombocytes (RSP) will be the default, and patients will then be included in that study arm. During day shifts, maximally two patients are included every 2 h, and during the night shift, maximally one patient is included.

### Preparation of DTC

**Fig. 2:**
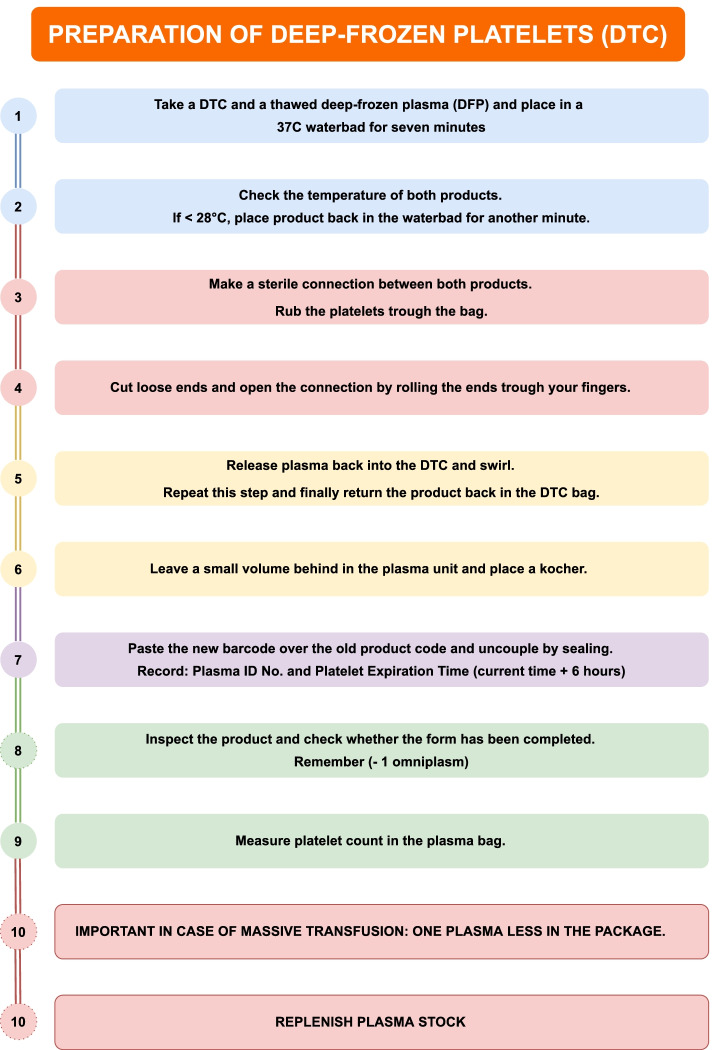
Preparation of deep-frozen platelets

The product characteristics of DTC and RSP are summarized in Table 2. The preparation flowchart for laboratory workers is shown in Fig. 2. Two units of thawed DFP are kept in cold storage (4 °C, maximum stored for 7 days) to enable rapid preparation of thawed DTC. After randomization to the DTC group, both a cold-stored thawed DFP and a DTC are placed in a 37 °C water bath for 7 min. Subsequently, both products are checked for leakage, temperature of both products > 28 °C by an infrared thermometer and visual abnormalities. If products are compliant but not above 28 °C, they are returned to the water bath for additional 1 min. Hereafter, the products and the warm plasma are added to the platelet bag while manually swirling the platelet bag to resuspend the platelets. In total, the preparation of a plasma-resuspended thawed DTC takes approximately 15 min. Immediately after the request of the DTC, the thawed DFP 4 °C stock is re-supplemented to a total of two units by thawing DFP (± 35 min).

**Table 2:** Characteristics of both platelet products

	RSP	DTC
Storage temperature	+ 22 °C	− 80 °C
Shelf life		
Deep-frozen	n.a	4 years
Thawed	7 days	6 h
Availability	Direct	15 min
Clot formation (in vitro, in vivo rat) [12]		Accelerated
Clot firmness (in vitro) [12]		Decreased
Aggregation (in vitro) [12]		Decreased
Organ injury (in vivo rat) [12]		Equal

*RSP* room temperature stored platelets, *DTC* deep-frozen platelets, *n.a* not applicable [12]

All participating hospitals work with a two-step MTP (two RBCs and two plasma units in the first MTP package) using these products from 4 °C liquid stock. For the second MTP package, the required plasma is thawed and is available 30 min after the first package is issued. The 15 min preparation time of DTC for the second MTP package will therefore not result in an additional delay compared to the RSP group.

To prevent different volumes of plasma being used in treatment (Omniplasma is the FFP used in The Netherlands and contains 200 ml of plasma), the MTP package in the DTC group contains one unit less plasma (because DTC already contains 300 ml plasma whereas the RSP are stored in 100 ml plasma and 200 ml additive solution).

### Preparation of RSP

In the participating centres, RSPs are readily available for transfusion from 22 °C liquid stock. After randomization to the control group, laboratory workers will take an RSP from storage and place it subsequently in the second MTP package. Despite the rapid availability of RSPs, the thawing time of plasma is the time-consuming factor in this study arm.

### Blinding

Blinding of caretakers is not possible; the blood component differs in product labelling which contains the name and code of the product. Furthermore, bag shape and texture, product volume and colour of the product differ. Bias of caretakers is reduced as much as possible by adhering to the same transfusion and treatment policies in both study arms. The bias of the researchers is reduced as much as possible by blinding the group allocation and only releasing the group allocation after the statistical analysis has been performed.

### Data management

All data will be stored in Castor EDC (Castor Research Inc.) All data are coded and are stored in accordance with the current regulations and will be handled confidentially. The handling of personal data will comply with the EU General Data Protection Regulation and the Dutch Act on Implementation of the General Data Protection Regulation.

### Statistical methods

Statistical analysis will be performed by using SPSS. The distribution of continuous data is assessed by the Shapiro-Wilk test. Normally distributed data will be expressed as mean with range and not normally distributed data with median and interquartile range. Homogeneity of variances will be tested with Levene’s test. A *p*-value < .05 is therefore considered as statistically significant. Patients will be analysed on a per-protocol base. Missing data are not expected for the primary outcome measure time to haemostasis. If missing data for other parameters are present, the predictive mean of the other values within the arm will be used.

Subgroup analysis will be performed for type of injury (blunt/penetrating/severe traumatic head injury (AIS ≥ 3) and type of surgery (trauma/vascular), and the patient received ≥ 10 units of erythrocyte concentrates/24 h. Categorical data will be analysed with the chi-squared test. Numbers and frequencies will be calculated and reported. Multivariate logistic regression analysis will be performed on the primary outcome measure. Based on 71 patients in each group, a total of four parameters will be included in the multivariate logistic regression. The utilization of DTC will be an independent variable in this model. Haemostasis (yes/no) will be the dependent variable. Covariates will be age, gender, type and severity of injury.

For repeated measures or longitudinal data (laboratory parameters, vital parameters, coagulation parameters and total in-hospital transfused blood component consumption), mixed measures ANOVA is used where groups are used as between-subjects factors.

Independent variable time (e.g. measurement one, two or three in rotational thromboelastometry (ROTEM)) will be used as the within-subjects factor. To investigate sphericity, Mauchly’s test of sphericity is used. If both corrections lead to the same conclusion, Greenhouse-Geisser will be used. If both Greenhouse-Geisser and Huynh-Feldt show different conclusions (e.g. Greenhouse-Geisser shows no effect (*p* > .05) and Huynh-Feldt shows the opposite (*p* < .05)), the mean of the two *p*-values is used. For mortality, a 30-day Kaplan-Meier survival curve will be computed. Cox proportional hazards model will be used to adjust for baseline covariates such as age, gender and injury severity based on injury severity score. A detailed statistical analysis plan including the main and secondary study parameters/endpoints is added in Additional file 2.

### Interim analysis

To reveal the large differences between the treatment groups which could lead to termination of the study, an interim analysis will be performed for the primary outcome measure after the inclusion of 20 patients. An alpha spending function of 0.06 with *t* = 25% with sample *n* = 20 and total *n* = 158 ((40/158) × 0.25) is used to correct significance. A *p*-value of < .003 will be considered as statistically significant. Numbers and frequencies will be calculated and reported. During the interim analysis, the inclusion of subjects will be continued. The trial will be terminated early if clear benefit or harm of the treatment is shown after the interim analysis. The primary outcome measure percentage of patients who achieved haemostasis will be analysed with the chi-squared test to assess the differences between the groups.

### Data monitoring and harms

The known burden and risks are not greater than the currently used blood platelets and serious adverse events as a result of platelet transfusion have not been reported [7, 9, 10]. Moreover, although a non-inferiority design is chosen, it is hypothesized that patients who received DTC achieve haemostasis earlier. As the administration of DTC instead of standard platelet concentrates is considered low-risk, no data safety monitoring board is installed. Monitoring will be performed annually by the Erasmus MC.

### Protocol amendments

A ‘substantial amendment’ is defined as an amendment to the terms of the medical ethical committee application (MEC), or to the protocol or any other supporting documentation, that is likely to affect to a significant degree on (1) the safety or physical or mental integrity of the subjects of the trial, (2) the scientific value of the trial, (3) the conduct or management of the trial or (4) the quality or safety of any intervention used in the trial. All substantial amendments will be notified to the MEC and to the competent authority. Non-substantial amendments will not be notified to the accredited MEC and the competent authority but will be recorded and filed by the sponsor.

### Consent or assent

The major part of the target population is not able to provide informed consent (e.g. intubated on arrival). The need for urgent treatment is high, and since it is not possible to await consent from a legal representative, ‘deferred consent’ will be requested as soon as possible. When patients or legal representatives remain unable to provide consent, after 30 days or decease, all collected data will be used. It is possible to resign from participation at any time without further explanation. We will request consent when possible, for the review of participants’ medical records and for the collection of blood samples to assess laboratory and coagulation parameters.

### Confidentiality and access to data

All included patients will receive a code that is related to the individual patient. Data can only be traced back to the individual patient using this code and the hospital patient number. This code is made up of a trial acronym and a unique patient ID (based on the randomization number in the envelope) (e.g. MAFOD-001). Data are stored for 15 years after the completion of the study. Only the principal investigator and coordinating investigator are able to identify the original hospital patient numbers both electronic and on paper. Data can only be traced back to the individual patient by members of the research team or the health inspector. Only the research team will have access to the final datasets. The informed consent documents and the datasets used and/or analysed during the current study will be available from the corresponding author upon reasonable request.

### Ancillary and post-trial care

Participating centres and included patients will not receive financial compensation for participation in the study. The hospital insurance provides cover for damage to research subjects through injury or death caused by the study. The insurance applies to the damage that becomes apparent during the study or within 4 years after the end of the study.

### Dissemination policy

Blinding is revealed after the analysis. Research data will be published in peer-reviewed journals and presented at scientific congresses and meetings. All data will be published anonymously and cannot be traced back to individual patients. Data will be announced 6 months after the termination of inclusion.

## Discussion

Despite the shown efficacy of its use in the military setting, deep-frozen or cryopreserved platelets have not been implemented or investigated widely in the civilian treatment of bleeding. The NLAF have been using DTC for the past 20 years. An animal study including rats with traumatic haemorrhage who were subsequently transfused with DTC showed reduced blood component use compared to RSP to reach and maintain a mean arterial pressure of 60 mmHg [11, 12]. In thrombocytopenic patients, the use of cryopreserved platelets appears to be safe and effective, and also in trauma [12], cardiopulmonary surgery [9] or thrombocytopenia [10, 14], the outcome of treatment with frozen platelets was similar to room-stored platelet transfusion. Despite this fact, high-quality evidence from randomized clinical trials for the efficacy of DTC in acute bleeding is still lacking.

Retrospective data from missions from the NLAF show that increased DTC and plasma use as a result of the implementation of an MTP resulted in a significant improvement in chances of survival during armed conflict [7]. However, in this setting, no RSP were available to provide evidence of an improved haemostatic potential of DTC in the treatment of acute bleeding, as has been suggested by the above-mentioned rat study [12] and one cardiopulmonary bypass surgery study [9]. We believe that DTCs provide a suitable and potentially better alternative to RSP in the treatment of bleeding patients. Therefore, we chose to initiate the first randomized controlled study to compare DTCs with RSPs during the first 48 h of the in-hospital treatment of traumatic and vascular surgical bleeding sufficient to trigger the activation of the MTP. We aim to show non-inferiority compared to RSP as we expect that platelet transfusion is only a small part of the treatment of severely injured patients with massive haemorrhage. In addition, the results may help to improve platelet transfusion policy during resuscitation management in general, and it may facilitate the implementation of these improved policies and or DTC in both civilian and military settings.

### Trial status

Protocol version 1.0 dated January 01, 2021. By August 01, 2022, the trial had not yet recruited patients. The estimated start of recruitment will be January 01, 2023. The recruitment is expected to continue for at least 24 months.

## Supplementary Information

**Additional file 1. **SPIRIT Checklist for *Trials.*

**Additional file 2.** Statistical analysis plan.

**Additional file 3.** Translation of the ethics approval.

## Data Availability

Only the principal investigator, coordinating investigator and project leaders are able to identify the original patient numbers both electronic and on paper.

## Abbreviations

DFP: Deep-frozen plasma
DTC: Deep-frozen thrombocytes
FFP: Fresh frozen plasma
FWB: Fresh whole blood
ITACTIC: Viscoelastic haemostatic assay augmented protocols for major trauma haemorrhage
MAFOD: MAssive transfusion of Frozen bloOD
MMB: Military blood bank
MEC: Medical ethical committee application
MTP: Massive transfusion protocol
NLAF: Netherlands Armed Forces
RBC: Red blood cells
ROTEM: Rotational thromboelastometry
RSP: Room temperature-stored platelets
SPIRIT: Standard Protocol Items: Recommendations for Interventional Trials
