# Supplementary material for: The haemostatic effect of deep-frozen platelets versus room temperature-stored platelets in the treatment of surgical bleeding: MAFOD—study protocol for a randomized controlled non-inferiority trial
Source: Trials. 2022 Sep 24;23:803. doi: 10.1186/s13063-022-06739-2 (PMC9509541; doi:10.1186/s13063-022-06739-2)
Supplement: Supplementary file 2 — Additional file 2. Statistical analysis plan. [file 13063_2022_6739_MOESM2_ESM.docx]

**Additional file 2**

**Statistical Analysis Plan**

**Main study parameters / endpoints**

Percentage of patients achieved haemostasis

Achieved haemostasis (yes/no) as defined a patient no further require erythrocyte transfusions for two hours;

**Secondary study parameters / endpoints**

1. **Time to haemostasis** as defined as the time in minutes from arrival to the moment a patient received no further erythrocyte transfusions for two hours;
2. **Total in hospital transfused blood component consumption** in different time intervals measured from time of inclusion (EC, plasma, platelets). Measurement in 0-1 and 1-3 and 3-6 and 6-12 and 12-24 and 24-72 hours and 72-hours – 30 days or discharge

- MTP activation? (yes / no)
- Time to first MTP (minutes)
- Time to first MTP with platelets request (minutes)
- Time to first blood component transfusion (minutes)
- Time to last blood component transfusion (minutes) in first 24 hours
- Amount of Crystalloids: in the ED, during surgery, ICU, total in 24 hours (units) (mL transfused)
- Amount of Colloids: in the ED, during surgery, ICU, total in 24 hours (units) (EC, plasma, platelets) (mL transfused)
- Type of plasma (omniplasma/Qplasma/both/unknown)
- Type of platelets (platelet additive solution (PAS) / plasma / both)
- Dose in blood products: (platelets, plasma: fibrinogen deep-frozen platelets (0.9 g/300ml); omniplasma )(0.6 gr/200ml); room temperature stored platelets (0.4 g/ 400ml): dose given in emergency department (ED), during surgery, during ICU stay and until discharge;

1. **Fibrinogen administration** in different time intervals measured from time of inclusion. Measurement in 0-1 and 1-3 and 3-6 and 6-12 and 12-24 and 24-72 hours and 72-hours – 30 days or discharge

- Fibrinogen Concentrate administered: (yes/no);
- Amount of fibrinogen concentrate administered at 0-1 and 1-3 and 3-6 and 6-12 and 12-24, 24-72 hours and total
- Calculated fibrinogen transfused with plasma and platelet units

1. **Laboratory parameters** in different time intervals measured from time of inclusion. Measurement at 0-1 and 1-3 and 3-6 and 6-12 and 12-24 and 24-72 hours

- haemoglobin (mmol/L);
- haematocrit (L/L);
- platelet count (x 109 / L);

1. **Coagulation parameters in different time intervals** in different time intervals measured from time of inclusion.

- Standard coagulation tests first measurement at 0-1 and 1-3 and 3-6 and 6-12 and 12-24 and 24-72 hours
  - - fibrinogen (clauss) (g/L);
    - INR;
    - aPTT (seconds)
- Presence of coagulopathy (defined as APTT >40 seconds and/or INR >1.2 and/or a platelet count <120*109 per litre) [57]
- Viscoelastic testing (ROTEM: measured by viscoelastic testing Extem, Intem, Fibtem, Heptem, Aptem
  - - Clotting time (seconds)
    - Clot formation time (seconds)
    - Maximum clot firmness
    - Amplitude 5/A5
    - Amplitude 10/A10
    - Amplitude 20/A20
    - Lysis after 30 minutes

1. **In-hospital survival**

- Alive at discharge (yes/no)
- Alive after ED (yes/no)
- Alive after surgery (yes/no)
- Time of death (hours:minutes)
- Location of death (free text)
- Cause of death
  - - Obduction (yes/no)
    - Time to death calculated from hospital arrival (hours:minutes)
    - Exsanguination: death caused by uncontrolled bleeding
    - Haemorrhagic shock: shock associated with the sudden and rapid loss of significant amounts of blood
    - Traumatic brain injury: an injury to the brain caused by penetration of the skull or movement of the brain within the skull
    - Respiratory/pulmonary contusion / tension pneumothorax: any loss of ventilatory capability, usually from a mechanical issue
    - Sepsis: an overwhelming systemic response to the documented infection.
    - Multi Organ Failure (MOF): altered organ function in at least two organ systems. Progressive and profound organ dysfunction that is incompatible with life.
    - Stroke: New neurological deficit not present prior to injury which is sudden or rapid in onset
    - Myocardial infarction: acute, irreversible myocardial injury
    - Pulmonary embolism: a blood clot lodged in the lumen of a pulmonary artery acutely causing death
    - Transfusion related fatality: fatality as a direct result of a complication of blood component transfusion

1. **Hospital length of stay**

- Number of days in hospital after admission (date of discharge minus date of admission to the hospital)
- Discharge location (home / other hospital / morgue / medical rehabilitation / other)

1. **ICU stay**

- Length of stay (days) calculated from ICU admission (date) to discharge (date)
- ICU re-admission (yes/no)
- ICU re-admission, discharge (date)
- Detubation (date)
- Ventilation free period (days from hospital admission)
- Apache II score (at admission, 24-hour after admission) (see attachments)
- MOF (at admission, 24-hour after admission) as defined by the Denver score (see attachments)
- Acute Kidney Injury (based on serum creatinine)

1. **Occurrence of transfusion reactions**

- Fever (> 2°C raise of temperature) / shivering (yes/no/unknown)
- Urticaria (yes/no/unknown)
- Respiratory distress (e causa ignota?) after transfusion (yes / no / unknown)
- Transfusion related lung injury (yes/no/unknown)
- Circulatory overload (yes/no/unknown)
- Hypotension direct after transfusion (yes / no / unknown)

### Other study parameters (if applicable)

**Baseline characteristics**

- age (years)
- gender (male/female)
- Blood group (A/B/AB/O/Unknown)
- Rhesus factor (-/+)
- Relevant medical history
  - Cardiovascular disease
  - Lung disease (e.g. chronic obstructive pulmonary disease)
  - Cerebral vascular accident
  - Previous traumatic injury
  - Diabetes
  - Malignancy
- Body mass index

**Injury severity (for trauma patients only)**

- Bleeding Score (ABC) based on:
  - Pulse ≥ 120 beats / minute
  - SBP ≤ 90 mm HG
  - Positive FAST (Focused Assessment of Sonograhy for Trauma)
- Severe traumatic brain injury (TBI) Abbreviated Injury Scale > 3
- Shock Index (SI), defined as SBP / HR
- Glasgow Coma Scale (3-15, unknown)
- Injury related: mechanism of injury (any blunt/penetrating/other)
- Injury severity:
  - Injury Severity Score (ISS) based on (head / face / neck / thorax / abdominal and pelvic / spine / upper extremity / lower extremity / external).
  - Revised Trauma Score requested from the Dutch National trauma Registry (LTR)
- Presence of coagulopathy on arrival (defined under 8.1.2, heading 5) (yes/no)
- Early laparotomy / thoracotomy (< 1 hour), (yes/no)

**Characteristics (for transplantation patients only)**

- Model for End-Stage Liver Disease (MELD) score
- Previous liver transplant (%)
- Serum bilirubin

**Prehospital characteristics (for trauma patients only)**

- - Self-referral (yes/no)
  - Helicopter Emergency Medical Service (HEMS) ordered? (yes/no)
  - Time of alarm, arrival at patient, arrival at hospital (hour:minute)
  - Transport vehicle (ambulance / helicopter / other (e.g. own transport / police car)
  - prehospital erythrocytes (yes/no/unknown)
  - prehospital erythrocytes (units transfused)
  - prehospital plasma (yes/no/unknown)
  - prehospital plasma (units transfused)
  - Prehospital crystalloids (yes/no/unknown)
  - Prehospital crystalloids (mL)
  - Vitals at T1, T2 and T3 during ambulance / HEMS transport (yes/no) (retrieved from ambulance or HEMS forms)
    - Heart Rate (HR) (beats per minute)
    - Systolic Blood Pressure (SBP) (mmHg)
    - Diastolic Blood Pressure (DBP) (mmHg)
    - Respiratory Rate (RR) (breaths per minute)
    - sPO2 (%)
    - Temperature (°C)
  - Tranexamic acid (yes / no / unknown);
  - Fibrinogen concentrate (yes / no / unknown)
  - Fibrinogen concentrate (gram)
  - Echo positive for bleeding (yes / no / unknown)
  - Estimated blood loss (mL)
  - Surgical intervention (*e.g.* thoracotomy) (yes / no / unknown) (specify)
  - Drain placement (yes/no)
  - Intubation? (yes / no)
  - Cardiopulmonary Resuscitation (yes/no), return of spontaneous circulation (yes/no), time of return of spontaneous circulation (minutes after HEMS/Ambulance alarm)

**Emergency Department (for trauma patients only)**

- FAST positive or proven bleeding CT (yes/no/unknown)
- Use of cell saver (yes/no)
- Relevant history of anticoagulant use (yes/no/unknown)
- Initial vital parameters (as described under vital parameters)
- First laboratory parameters as described under laboratory parameters after arrival
- Drain placement (yes/no)
- Estimated blood volume in drain (mL)

**Operating room characteristics**

- Surgery (yes/no)
- Time to first operation (minutes)
- Duration of surgery (minutes) calculated from start to end of surgery
- Estimated blood loss (mL)
- Additional surgery after first surgery (yes/no) (specify)
- Time to second surgery (minutes)
- Duration of second surgery (minutes)
- Additional blood product use during second surgery (units EC, plasma, platelets)
- Number of surgical procedures during hospital stay (number)
- Type of surgery (e.g. damage control surgery or definitive surgery)
- Venovenous bypass (%) (**for transplantation patients only)**

**Additional medication administered**

- Tranexamic acid: administered (yes / no); time to first gift (minutes); dose given in between categories in intervals: 0-1 and 1-3 and 3-6 and 6-12 and 12-24 and 24-72 hours
- Prothrombin Complex Concentrate: administered (yes/no); 0-1 and 1-3 and 3-6 and 6-12 and 12-24 and 24-72 hours
- Calcium gluconate: administered (yes/no); time to first gift (minutes); dose given in between categories in intervals: 0-1 and 1-3 and 3-6 and 6-12 and 12-24 and 24-72 hours
- NoVo seven (recombinant factor VIIa): administered (yes/no);
- Idarucizumab: administered (yes/no)
- Protamine: administered (yes/no)
- Bicarbonate: administered (yes/no)
- Heparin/thromboprophylaxis (yes/no)
- Heparin/thromboprophylaxis: time to first administration;
- Specific medical treatment to counteract platelet antagonist and/or plasma anticoagulants (yes/no, if yes specify)

**Laboratory parameters** (first measurement after inclusion at 0-1 and 1-3 and 3-6 and 6-12 and 12-24 and 24-72 hours

- - Glucose (mmol/L)
  - Sodium (mmol/L)
  - Potassium (mmol/L)
  - Chloride (mmol/L)
  - Calcium (mmol/L)
  - pH
  - pCO2 (kPa)
  - pO2 (kPa)
  - Active bicarbonate (mmol/L)
  - Standard bicarbonate (mmol/L)
  - Base Excess (BE) (mmol/L)
  - Lactate (mmol/L)
  - Serum creatinine
  - Serum billirubin

**Vital parameters** (first measurement after inclusion at 0-1 and 1-3 and 3-6 and 6-12 and 12-24 and 24-72 hours

- Vitals available (yes/no)
- Heart Rate (HR) (beats per minute)
- Systolic Blood Pressure (SBP) (mmHg)
- Diastolic Blood Pressure (DBP) (mmHg)
- Respiratory Rate (RR) (breaths per minute)
- sPO2 (%)
- Temperature (°C)

**STATISTICAL ANALYSIS**

Statistical analysis will be performed by using SPSS version 25 or higher. Distribution of continuous data is assessed by Shapiro-Wilk test. Homogeneity of variances will be tested with Levene’s test. A p-value < 0.05 is considered as statistically significant. Patients will be analysed on a per protocol base. Patients who deceased during the treatment will also remain in the analysis. Missing data are not expected for the primary outcome measure time to haemostasis. If missing data for other parameters are present, the predictive mean matching is used to predict the target value using 5 subjects.

**Subgroup analysis**

- Patients with blunt traumatic injury
- Patients with penetrating injury
- Patients with severe traumatic head injury (AIS ≥ 3)
- Patients received ≥ 10 units of erythrocyte concentrates / 24 hour
- Transplantation surgery
- Trauma patients

## Primary study parameter(s)

Categorical data will be analysed with Chi-squared test to assess differences between groups. Numbers and frequencies will be calculated and reported. To investigate the possible effects of confounders, multivariate analysis will be performed on the primary outcome measure. Achieved haemostasis will be defined as dependent variable. Independent variables will be baseline characteristics age, gender and injury severity.

## Secondary study parameter(s)

Continuous data will be reported with mean and SD (if normal distributed) or with median and quartiles (25^th^-75^th^) (if not normal distributed). Data will be analysed with a Student’s t-test (if normally distributed) or with a Mann-Whitney U-test (if not normal distributed). Categorical data will be analysed with Chi-squared test or the Fisher’s exact as applicable. Numbers and frequencies will be reported.

For outcome measures measured over time, linear mixed-model analysis over time (fixed effects: treatment and covariables baseline characteristics such as age). Comparison between time points will be adjusted for multiple testing using the method of Sidak. The significance of the correlation from this mixed-model analysis and the random effect for predicting the outcome measure is assessed as p ≤ 0.005.

**Mortality**

Mortality will be computed at 1, 3, 6, 12, 24 and 72 hours and 30-days. A 30-day Kaplan-Meier survival curve will be computed. Cox regression will be used to take random effects into account. The analysis for morality will be adjusted for demographic variables age, gender and injury severity based on ISS. It is imaginable that patients decease prior to receive any blood product. All patients will included in primary analysis as randomized.

## Other study parameters

For patient characteristics, descriptive analysis will be performed for both groups (control treatment versus intervention treatment). Continuous data will be reported with mean and SD (if normal distributed) or with median and quartiles (25^th^-75^th^) if not normally distributed. Numbers and frequencies are used to report categorical data. Categorical data (e.g. gender) will be analysed with Chi-squared test. Continuous data (e.g. age) will be reported with mean and SD (if normal distributed) or with median and quartiles (25^th^-75^th^) if not normal distributed. Massive transfusion in the subgroup ‘other specialisms’ will only be narratively described.

## Interim analysis (if applicable)

Interim analysis will be performed for the primary outcome measure after the enrolment of 20 patients. During the interim analysis, the inclusion of subjects will be continued. Trial will be terminated early due to clear benefit or harm of the treatment. Primary outcome measure percentage of patients achieved haemostasis will be analysed with Chi-squared test to assess differences between groups. A p value of < 0.05 will be considered as statistically significant. Numbers and frequencies will be calculated and reported. Interim analysis will be performed by a statistician of the Ministry of Defence / Alrijne Ziekenhuis Leiderdorp and results will be reported to Erasmus MC, the principal investigator, the coordinating investigator and the project leaders.
