# Supplementary material for: The haemostatic effect of deep-frozen platelets versus room temperature-stored platelets in the treatment of surgical bleeding: MAFOD—study protocol for a randomized controlled non-inferiority trial
Source: Trials. 2022 Sep 24;23:803. doi: 10.1186/s13063-022-06739-2 (PMC9509541; doi:10.1186/s13063-022-06739-2)
Supplement: Supplementary file 3 — Additional file 3. Translation of the ethics approval. [file 13063_2022_6739_MOESM3_ESM.docx]

**Additional file 3 Translation of the Ethics approval**

Title of study:

The haemostatic effect of thawed deep-frozen platelets resuspendedin thawed plasma versusroom temperature stored platelets in platelet additive solution in the treatment of surgical bleeding (MAFOD); a randomized controlled non-inferiority trial. (MAFOD)’

Dear dr. Hoencamp,

The Erasmus MC Medical Ethics Review Committee (METC) has approved the above research proposal (fully in accordance with the requirements of the METC) received on March 19, 2021.

Decision

The committee has approved the research proposal within the framework of the medical scientific research with people (WMO) discussed in the 1270^e^ meeting d.d. 20 April 2021. As a result of this assessment, the committee has asked you a few questions or comments submitted. The questions and comments are satisfactory answered.

The committee judges **positive** about the execution ing of the research in the Erasmus MC.

Before you can start carrying out the research at Erasmus MC, you must: to be in possession of permission to conduct the research by the Department head. The committee does not need to receive a copy of this. WMO test pressmisinsurance The test persons included in this study fall under the Erasmus MC test subjects insurance. Report the following information must be reported to the committee: Start date (date on which the first subject is included); end of inclusion (date the last subject was enrolled); end date (date on which the last measurement in the last subject has occurred)

Submit for review

The following documents must be submitted to the commission to be submitted for assessment:

Unexpected and serious side effects via portal ToetsingOnline.Amendments and/or addenda

Form Annual progress report, for the first time one year after this decision

Publications and final report

BROK course further wise the committee advises the researchers that, in accordance with national agreements, clinical researchers are required to take the “Basic course on regulation and organization of clinical research” (BROK) and obtain the corresponding certificate. The committee will don’t assume that, if this is not the case by now, the clinical investigators of this study will not exceed 6 have fulfilled this obligation months after the start of the study. For information

About the BROK

Finally, I would like to point out that pursuant to Article 23 of the Medical Act scientific research with people in conjunction with article 7:1 of the General Administrative Law Act, the person whose interest is directly involved in this decision against it within six weeks after the day when

this decision has been made known, can submit an administrative appeal to the Central Office

Human Research Committee. The committee has the power to withdraw its positive decision if it is established that the conduct of the research is seriously deficient. The positive decision of the committee loses validity as with the execution of the research has not started inside two year after this decision has been made.

Sincerely,

On behalf of the Erasmus MC Medical Ethics Review Committee,
